# Supplementary figures and images for: Occurrence of L1M Elements in Chromosomal Rearrangements Associated to Chronic Myeloid Leukemia (CML): Insights from Patient-Specific Breakpoints Characterization
Source: Genes (Basel). 2023 Jun 27;14(7):1351. doi: 10.3390/genes14071351 (PMC10379433; doi:10.3390/genes14071351)

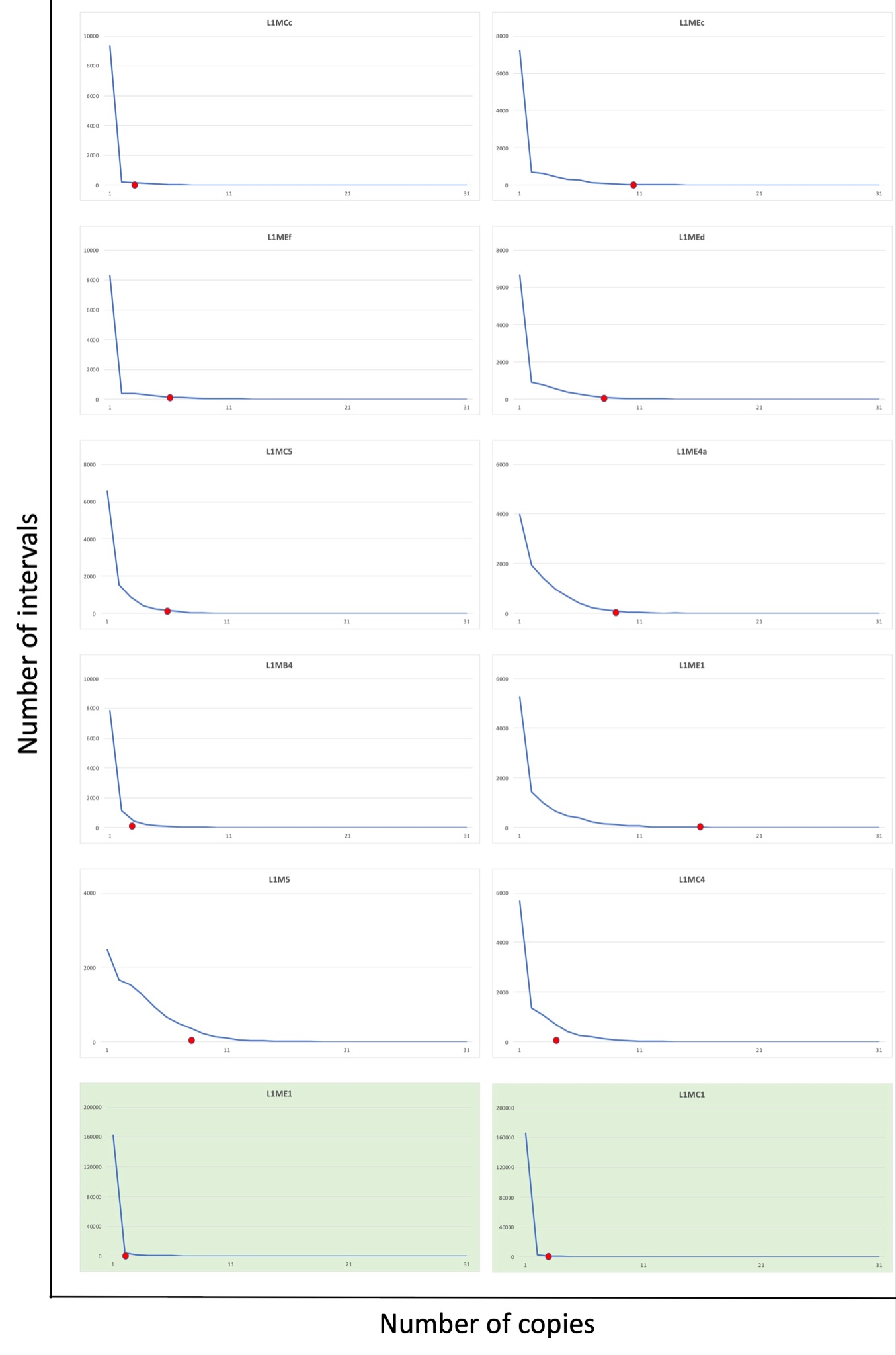

Supplement: Supplementary file 1 [file genes-14-01351-s001.zip › Supplementary Files/Figure S1 Occurrence of L1M elements in chromosomal rearrangements associated to Chronic Myeloid Leukemia (CML)- insights from patient-specific breakpoints characterization.tif]
